# Novel Strategy for Selection of Monoclonal Antibodies Against Highly Conserved Antigens: Phage Library Panning Against Ephrin-B2 Displayed on Yeast

**DOI:** 10.1371/journal.pone.0030680

**Published:** 2012-01-23

**Authors:** Xiaoling Gu, Yogindra Vedvyas, Xiaoyue Chen, Tanwi Kaushik, Chang-Il Hwang, Xuebo Hu, Alexander Y. Nikitin, Moonsoo M. Jin

**Affiliations:** 1 Department of Biomedical Engineering, Cornell University, Ithaca, New York, United States of America; 2 Department of Biomedical Sciences, Cornell University, Ithaca, New York, United States of America; University of California Merced, United States of America

## Abstract

Ephrin-B2 is predominately expressed in endothelium of arterial origin, involved in developmental angiogenesis and neovasculature formation through its interaction with EphB4. Despite its importance in physiology and pathological conditions, it has been challenging to produce monoclonal antibodies against ephrin-B2 due to its high conservation in sequence throughout human and rodents. Using a novel approach for antibody selection by panning a phage library of human antibody against antigens displayed in yeast, we have isolated high affinity antibodies against ephrin-B2. The function of one high affinity binder (named as ‘EC8’) was manifested in its ability to inhibit ephrin-B2 interaction with EphB4, to cross-react with murine ephrin-B2, and to induce internalization into ephrin-B2 expressing cells. EC8 was also compatible with immunoprecipitation and detection of ephrin-B2 expression in the tissue after standard chemical fixation procedure. Consistent with previous reports on ephrin-B2 induction in some epithelial tumors and tumor-associated vasculatures, EC8 specifically detected ephrin-B2 in tumors as well as the vasculature within and outside of the tumors. We envision that monoclonal antibody developed in this study may be used as a reagent to probe ephrin-B2 distribution in normal as well as in pathological conditions and to antagonize ephrin-B2 interaction with EphB4 for basic science and therapeutic applications.

## Introduction

The erythropoietin-producing hepatocellular (Eph) receptors and their ligands, ephrins comprise the largest subfamily of receptor tyrosine kinases (RTK), playing an important role in physiology such as embryogenesis, organ development, and angiogenesis as well as implicated in several types of cancers [1]. Among different classes of ephrins, ephrin-B2 is primarily expressed in arterial endothelial cells and neovasculature, forming a bidirectional signal with its cognate receptor EphB4, which is mainly expressed in venous endothelial cell [2], [3]. The importance of such interaction in a developmental process has been demonstrated by impaired angiogenesis and ultimately embryonic lethality in mice due to homozygous mutation of ephrin-B2 or EphB4 [3], [4], [5], [6]. The role of EphB4 and ephrin-B2 also extends to tumor growth and angiogenesis [1], [7]. Inhibition of their interaction by EphB4 antibody or extracellular fragment of EphB4 can inhibit tumor angiogenesis and tumor growth [8], [9], [10]. Ephrin-B2 is involved in vascular endothelial growth factor (VEGF) signaling, through the internalization of VEGF receptor in all endothelial cell types during physiological and pathological angiogenesis [11], [12], [13], and could be upregulated in VEGF-treated endothelial cells [5], [6]. Expression of ephrin-B2 along with EphB4 was found to be higher in many tumors including colorectal, breast, ovarian, and lung, serving as a poor prognostic marker [14], [15], [16], [17], [18].

Despite the importance of ephrin-B2 in physiology and pathological conditions, there are no widely available monoclonal antibodies against ephrin-B2, likely attributed to the fact that immume system in rodents prevents responses to self antigen or to highly conserved human antigens. To overcome the problem with generating antibodies against highly conserved antigens, mice with impaired immune tolerance (e.g. NZB/W) have been exploited [19], [20]; however, concerns remain on this alternative approach due to the observations of multi-specificity and low-affinity on auto-antibodies developed from autoimmune mice [20]. In order to generate antibodies against highly conserved ephrin-B2, we used phage display of single chain human antibody and screened them against ephrin-B2 expressed in yeast. From our previous work [21], we found that phage panning against antigens displayed in yeast is highly efficient in rapid enrichment of specific phage clones, obviating the need to produce soluble antigens as well as ensuring native conformation. With newly developed monoclonal antibody, we found that tumors of colon, breast, ovary, and lung upregulated ephrin-B2 compared to respective normal tissues. Antibody staining was also observed in the neovasculature within the tumor, corresponding to new vessel sprouts. Our antibody also exhibited properties such as its ability to cross-react with murine ephrin-B2, to inhibit EphB4 binding, and to be internalized into cells after binding to ephrin-B2. We anticipate that antibodies developed in this study will be useful in probing ephrin-B2 distribution in normal and disease processes, and in antagonizing the interaction between ephrin-B2 and EphB4 for scientific and therapeutic applications.

## Results

### Novel strategy of selecting antibodies against ephrin-B2

**Figure 1:**
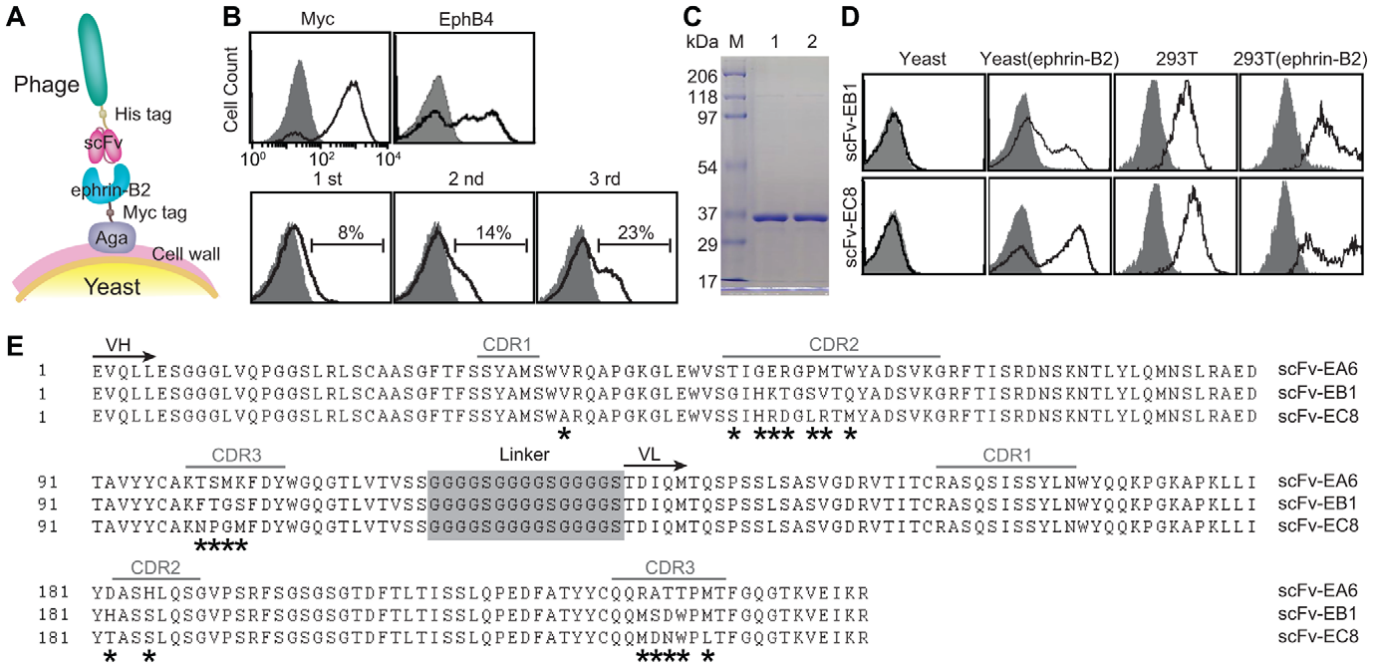
Selection, validation, and sequence of ephrin-B2-specific human single-chain antibodies. (A) A schematic diagram of phage panning against antigens expressed in yeast display system [34]. (B) Immunofluorescence flow cytometry measurements of protein and phage binding to yeast cells. Surface-displayed ephrin-B2 was detected by the binding of anti-Myc antibody (‘Myc’) as well as recombinant human EphB4-Fc (‘EphB4’) to yeast cells (top panel). Progressive enrichment of phage clones from first three rounds of panning (denoted as ‘1 st’, ‘2 nd’ and ‘3 rd’) was detected by antibody against His tag (bottom panel). Histograms drawn in shaded area and solid lines indicate antibody binding to uninduced and induced yeast cells, respectively. The percentage of phage clones with positive binding is indicated. (C) SDS-PAGE of scFv-EB1 (lane ‘1’) and scFv-EC8 (lane ‘2’). (D) Ephrin-B2 specific scFv binding to irrelevant yeast cells, yeast cells with expression of ephrin-B2 ectodomain, 293T cells, and 293T cells with transient expression of full-length ephrin-B2. Shown are the histograms of cells labeled with secondary antibody with (solid line) and without (shaded area) ephrin-B2 specific scFv as primary antibody. (E) Sequence alignment of scFv-EA6, scFv-EB1, and scFv-EC8. Complementarity determining regions (CDR), the beginning of immunoglobulin variable heavy (VH) and variable light (VL) chain domains, and the linker connecting VH and VL are noted. * indicates amino acids differ between scFvs.

We have previously shown that phage library of human antibody can be directly panned against antigens expressed in yeast (Fig. 1A) with great efficiency in selection of high affinity monoclonal antibodies [21]. Surface expression of ectodomain of ephrin-B2 on yeast cell surface was first validated by antibody binding to Myc tag, which was placed at the C-terminal of ephrin-B2, as well as the binding of EphB4, a physiological receptor of ephrin-B2 (Fig. 1A&B). Subtractive panning of a phage library of human single chain fragment variable fragment (scFv), consisting of depletion against yeast expressing irrelevant antigens followed by positive selection against ephrin-B2, resulted in a progressive increase in the percentage of phage clones bound to ephrin-B2 (Fig. 1B). A total of 96 phage clones were selected from the third round pool and tested individually for binding to ephrin-B2 using flow cytometry. This resulted in 85 clones with positive binding (data not shown). Out of these 85 clones, 10 high-affinity phage clones were then sequenced, which identified three unique clones (designated as scFv-EA6, scFv-EB1 and scFv-EC8). Two best binders, scFv-EB1 and scFv-EC8 were chosen for production in bacteria and purified by nickel-nitrilotriacetic acid (Ni-NTA) column, followed by gel filtration chromatography, which resulted in one distinct band around 35 kDa in SDS-PAGE (Fig. 1C). Soluble scFv-EB1 and scFv-EC8 not only retained binding to yeast but also showed specific binding to ephrin-B2 expressed in mammalian cells, judging from the detection of basal expression in 293T and enhanced binding after transient expression of full-length ephrin-B2 in 293T (Fig. 1D). scFv-EA6, scFv-EB1 and scFv-EC8 differed mainly within the complementarity determining regions (Fig. 1E). Overall, scFv-EC8 showed highest binding affinity to ephrin-B2, and was chosen for further studies.

### Characterization of single-chain antibody fused to Fc domain

**Figure 2:**
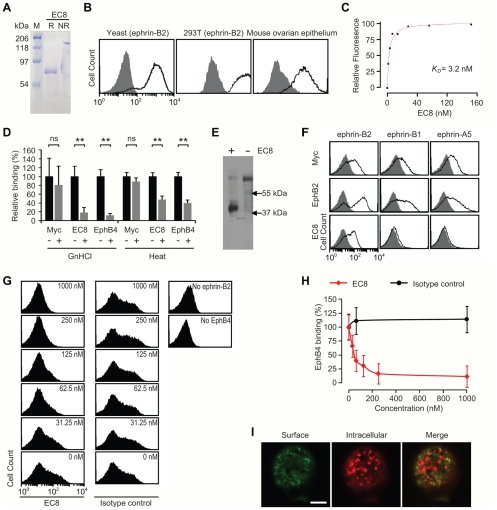
Conversion of scFv-EC8 as a fusion to immunoglobulin Fc and functional test. (A) SDS-PAGE images of EC8 resolved under reducing (R) and non-reducing (NR) conditions. (B) EC8 (solid line) binding to yeast cells, 293T with stable expression of ephrin-B2, and murine ovarian epithelium. The binding of isotype control is shown in shaded area. (C) Flow cytometry measurements of EC8 binding to 293T cells are shown in filled squares. First order Langmuir adsorption model was used to fit the data to estimate equilibrium dissociation constant (*K_D_*). (D) Conformation speficifity of EC8 against eprhin-B2 was examined by flow cytometry with (‘+’) or without (‘−’) incubating cells either in 6 M guanidine hydrocholoride (‘GnHCl’) for 20 min or in elevated temperature at 80°C (‘Heat’) for 10 min. (E) Western blot image of immunoprecipitated ephrin-B2 from 293T cells with (‘+’) or without (‘−’) EC8 antibody, detected by rabbit ephrin-B2 polyclonal antibody. (F) Flow cytometry measurements of EC8 binding to ephrin-B1 and ephrin-A5 displayed on yeast. Labeling of uninduced yeast cells is shown in shaded histograms. (G&H) Competition assay. Relative binding of EphB4 (100 nM) to yeast cells expressing ephrin-B2, preincubated with varying concentrations of EC8, was measured by flow cytometry. Affinity purified human IgG was included as isotype control. n = 3 independent measurements. (I) Confocal microscopic images of surface-bound EC8 and internalized ones before and after membrane permeabilization of 293T cells. Scale bar = 10 µm.

Although scFv-EC8 was fully functional in binding to ephrin-B2, for future *in vivo* applications and many standard assays relying on the presence of immunoglobulin Fc region, scFv fusion to Fc was constructed in pcDNA3.1 and produced in mammalian cells (Fig. 2A). Under the reducing condition, the Fc fusion of scFV-EC8 migrated around 64 kDa, consistent with its nominal molecular weight. scFv-EC8 fusion to Fc (designated as EC8) was fully functional, retaining specific binding to ephrin-B2 expressed in yeast and in 293T (Fig. 2B) with higher level of binding compared with the levels seen with scFv-EC8 (Fig. 1D). EC8 also exhibited comparable binding to murine ephrin-B2 expressed in transformed murine ovarian cells [22] (Fig. 2B), an anticipated result due to the fact that murine ephrin-B2 differed by only three residues from human ephrin-B2 expressed in yeast (Thr-22 to Gly-165) for phage screening. The binding affinity (equilibrium dissociation constant, *K_D_*) of EC8 to ephrin-B2 was around 3.2 nM, based on the fluorescence measurement using ephrin-B2-expressing 293T cells labeled in serial dilutions of EC8 (Fig. 2C). The increase in affinity of EC8 to ephrin-B2 was due to the dimerization effect of scFv, commonly noted as bivalency or avidity effect.

EC8 was conformation-specific against ephrin-B2, indicated from significant loss of binding to ephrin-B2 expressing yeast cells when ephrin-B2 was partially denatured by incubating cells either in guanidine hydrochloride at 6 M or in elevated temperature (Fig. 2D). The reduction in the level of EC8 binding to ephrin-B2 was not due to a change in surface expression as antibody binding to Myc tag was invariant. The property of EC8 to recognize conformation-specific epitope was also corroborated by its inability to stain ephrin-B2 in western blot (data not shown). We then tested if EC8 would specifically pull down ephrin-B2 from detergent-solubilized 293T cell lysates. Conformation-specificity of monoclonal antibody (mAb) EC8 was also confirmed with the detection of one distinct band of ∼45 kDa in size from the lysates only after precipitation with EC8, stained by polyclonal antibody against ephrin-B2 (Fig. 2E). Due to high sequence homology among some members of ephrin family, polyclonal antibodies against ephrin-B2 often crossreact with other ephrins like ephrin-B1 and ephrin-A5, which share sequence identity at 43% and 41% with ephrin-B2, respectively. While EphB2 showed comparable binding to all three ephrins tested, EC8 was found specific to ephrin-B2 with little binding to ephrin-B1 and ephrin-A5 (Fig. 2F).

### mAb EC8 blocked ephrin-B2 interaction with EphB4 and was internalized by ephrin-B2 ligation

After confirming specific binding of EC8 to ephrin-B2, we examined if EC8 was able to block ephrin-B2 interaction with its cognate receptor, EphB4. This would potentially be useful in blocking bidirectional signals triggered by ephrin-B2 and EphB4 interaction, which is important for normal physiology as well as in disease progression. In comparison to human immunoglobulin G (IgG) as isotype control, increasing concentration of EC8 added to yeast cells expressing eprhin-B2 led to a gradual decrease in the binding of EphB4 (used at 100 nM) to ephrin-B2, close to complete inhibition seen at 250 nM of EC8 (Fig. 2G&H). We detected the distribution of EC8 in 293T after labeling under fluorescence microscope, and observed that ephrin-B2 ligation by EC8 triggered cells to internalize the antibody. In order to differentiate surface-bound vs. intracellular pool of EC8, confocal microscopy was used to image cells prior to and after membrane permeabilization, which visualized distinct intracellular staining of EC8 (Fig. 2I).

### EC8 detection of ephrin-B2 expression in tumor tissues and tumor-associated vasculature

**Figure 3:**
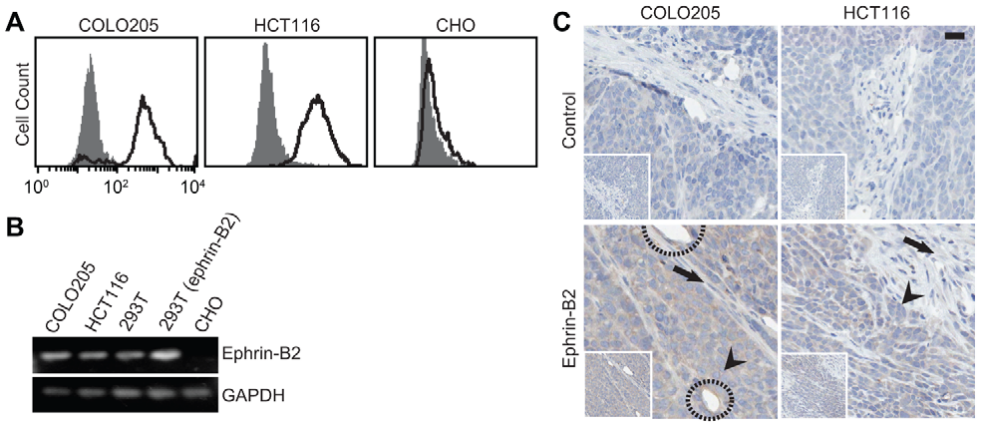
Detection of ephrin-B2 expression in human cancer cell lines and tumor xenograft in mice. (A) Flow cytometry measurements of EC8 binding to COLO205 and HCT116 cells (solid line) in comparison to the isotype control (shaded area). CHO cells with no ephrin-B2 expression were also included for comparison. (B) RT-PCR detection of ephrin-B2 expression in different cell lines. (C) Immunostaining of ephrin-B2 on human colon cancer xenografted in mice. Control denotes immunostaining without EC8 as a primary antibody. Tumor and stromal cells were indicated with arrowhead and arrow, respectively. Circle indicates murine endothelium stained with EC8. Scale bar = 20 µm.

Upregulation of ephrin-B2/EphB4 has been observed in many tumors, including ovary, colon, breast, and glioma, with a strong correlation with poor prognosis [14], [15], [16], [17], [18], [23], [24]. With two widely used colon cancer cell lines, COLO205 and HCT116, we found upregulation of ephrin-B2 by flow cytometry using EC8 (Fig. 3A). Chinese hamster ovarian (CHO) cells with no ephrin-B2 expression were included as a negative control. The relative levels of eprhin-B2 between different cell lines were further confirmed by RT-PCR, with 293T cells stably expressing ephrin-B2 as a positive control (Fig. 3B). Since not all conformation-specific antibodies are compatible with immunohistology after a standard procedure for tissue fixation, we examined if EC8 can be used to probe eprhin-B2 expression in the tissue. When colon tumor tissues were immunostained after tumor growth in mice and harvest, EC8 not only delineated human tumor xenograft with high ephrin-B2 expression, but also identified the vasculature within the tumor due to the cross-reactivity of EC8 with both human and murine ephrin-B2 (Fig. 3C). EC8 staining of ephrin-B2 clearly differentiated tumor cells (marked with arrowhead) from the murine stroma (marked with arrow) (Fig. 3C).

### EC8 detection of the upregulation of ephrin-B2 in diverse human tumors of epithelial origin

**Figure 4:**
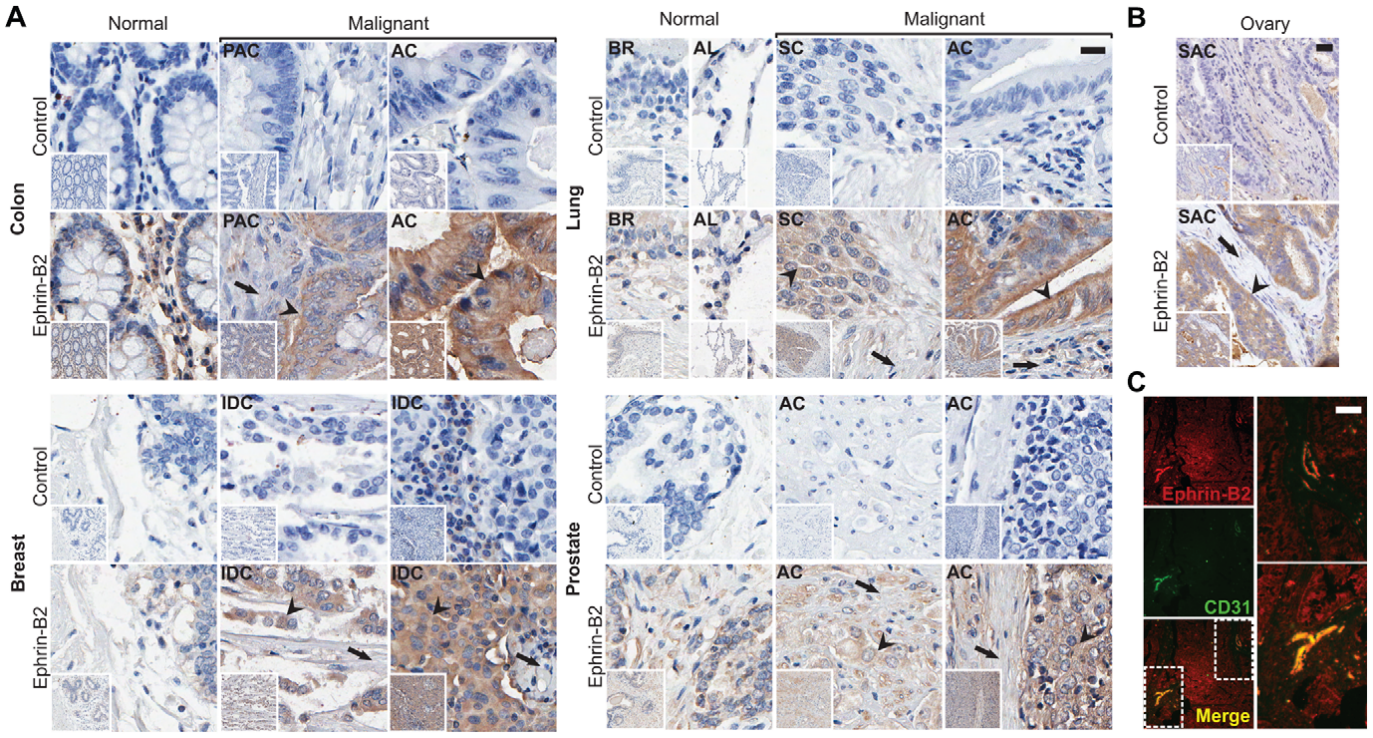
Detection of ephrin-B2 expression in human tissue arrays. (A&B) Immunostaining of ephrin-B2 expression in human tumor tissue arrays using EC8. Control denotes immunostaining without EC8 as a primary antibody. Tumor and stromal cells were indicated with arrowhead and arrow, respectively. Scale bar = 20 µm. PAC = Papillary Adenocarcinoma; AC = Adenocarcinoma; BR = Bronchus; AL = Alveoli; SC = Squamous Cell Carcinoma; SAC = Serous Adenocarcinoma. IDC = Nonspecific Infiltrating Duct Carcinoma. (C) Immunofluorescence staining on human colon tumor tissue demonstrating that EC8 (red) detects ephrin-B2 expressions in both cancer cells and tumor-associated vasculature (green). Blow up views of the two areas indicated with dashed box are shown in the right panel. Scale bar = 100 µm.

We further examined if EC8 could be applied to detect ephrin-B2 expression in other types of tumor directly collected from human patients. Among several epithelium-originated cancers, ranging from ovarian, lung, prostate, breast to colon, high level expression of ephrin-B2 was all found by immunostaining (Fig. 4A&B). In comparison to normal tissues or tumor stroma showing no or only baseline ephrin-B2 staining, malignant tumor cells characterized by their irregular cell shape (e.g. colon and lung) and aberrantly enlarged (e.g. colon, lung and prostate) or dense (e.g. breast) nucleoli had distinctively higher ephrin-B2 staining. Dual staining on human colon cancer sample with both anti-CD31 antibody and EC8 revealed some of the vasculatures that were co-stained, indicative of EC8 detecting tumor-associated vasculature (Fig. 4C).

## Discussion

Ephrin-B2 is preferentially expressed in arterial endothelium and smooth muscle cells, as well as neovasculature within the tumor. Expression of ephrin-B2 is modulated by VEGF, smooth muscle cell contact, and stress [25], [26], [27]. Ephrin-B2 has also shown to be upregulated in many cancers, including colon [15], uterine [16], ovarian [17] and esophageal cancers [18]. Despite the importance of the role of ephrin-B2 in physiology and disease, up until now monoclonal antibody specific to ephrin-B2 has not been widely available. This may be attributed to the fact that human ephrin-B2 is highly homologous to those of other mammals including rodents, presenting a challenge to isolating high affinity antibody from immunization and hybridoma technique. Limited *in vivo* alternatives for making antibodies against highly conserved antigens, including using mice with impaired immune response [19], [20], have been reported, yet the concerns remain on the multi-specificity and low-affinity of auto-antibodies [20].

Here we report the isolation of monoclonal antibody against ephrin-B2, which was selected from a phage library of human single chain antibody. Rather than panning phage clones against soluble antigens, we used yeast cells expressing antigens to pull down reactive phage clones, which was found to be highly efficient in rapidly enriching specific phage library. Given the library diversity (10^8^) and an enrichment factor of 10^2^–10^3^ per each round of our screening strategy [21], we were able to observe specific phage clones reactive to ephrin-B2 as early as after two rounds of sorting. As antibody selection is based on monomeric interaction between antigen-antibody, after conversion into a dimer by fusion to IgG Fc, the affinity (*K_D_* = 3.2 nM) of ephrin-B2 antibody (EC8) was comparable to those of high affinity monoclonal antibodies produced from hybridoma. Compared to many polyclonal antibodies generated from peptide fragments by immunization, EC8 selected against ephrin-B2 displayed in native conformation on yeast surface was found to be conformation-specific and discriminate ephrin-B2 from other ephrins with high homology.

Antibodies against EphB4 were found to inhibit tumor growth and angiogenesis, some of which are being investigated for anti-cancer therapy in preclinical studies [9], [10]. Eph receptor antibodies that were conjugated to small molecule drugs caused internalization of drugs and inhibition of tumor growth in vivo [28], [29]. Soluble extracellular domain of EphB4 targeting ephrin-B2 has been used in inhibiting angiogenesis and tumor growth in vivo [8]. We have found that mAb EC8 potently antagonized ephrin-B2 binding to EphB4, which would block forward and reverse signaling by EphB4 and ephrin-B2 interaction. Similar to the observation of EphB4 upregulation in some tumors, when human tissue array was probed with mAb EC8, ephrin-B2 was found to be also overexpressed in tumors in lung, breast, ovary, colon, and prostate over respective normal tissue. Consistent with the previous observation of ephrin-B2 expression in tumor-associated vasculature, EC8 delineated ephrin-B2 expression in newly formed vessels within the tumor. Upregulation of ephrin-B2 was also found in colorectal cancer cell lines, COLO205 and HT108 both in cell culture and as a tumor xenograft in mice. Notably, due to the cross-reactive nature of antibody with murine ephrin-B2, mAb EC8 also identified tumor-associated vasculature, simultaneously detecting ephrin-B2 in human tumor as well as ephrin-B2 in murine host.

Neovasculatures in adults sprouting from arterial vessels and capillaries, whether caused by VEGF-signaling, tissue injury, or tumor growth, were found to express ephrin-B2. It is unknown how ephrin-B2 upregulation in some of the tumors of epithelium origin would perturb the balance between ephrin-B2 and Eph4 expressed in arterial and venous vessels, respectively, and contribute to the tumor growth and metastasis. Given the observation that overexpression of ephrin-B2 in some tumors is correlated with poor prognosis [1], [16], it will be an interesting question if the role of ephrin-B2 together with EphB receptors in some tumor is associated with the promotion of the vasculature growth and the adenoma-carcinoma transition, facilitating tumor metastasis. We propose that high affinity and antagonist antibodies such as EC8 would provide a valuable tool for examining the role of ephrin-B2 expression on tumor angiogenesis and migration.

## Materials and Methods

### Selection and expression of ephrin-B2-specific scFvs

A part of human ephrin-B2 ectodomain (Thr-22 to Gly-165) was cloned into yeast surface display vector, CAga2 (a modified version of PCTCON [30]), and transformed into yeast strain EBY100 by EZ-transformation kit (Zymo Research). Yeast culture and protein induction were performed as previously described [30]. Proteins displayed on yeast contain ephrin-B2 ectodomain, Myc tag, and Aga2 from N- to C-terminal. Surface expression of ephrin-B2 in yeast was measured by immunofluorescence flow cytometry using mouse anti-Myc antibody (9E10, Santa Cruz Biotechnology) followed by goat phycoerythrin-conjugated anti-mouse antibody (Santa Cruz Biotechnology), as well as by recombinant human EphB4-Fc chimera (Biomiga) followed by goat phycoerythrin-conjugated anti-human antibody (Santa Cruz Biotechnology). A phage library of human single chain variable fragment (scFv) antibody with 10^8^ diversity (Tomlinson I+J, Source BioScience) was panned against ephrin-B2 expressing-yeast cells, following a procedure previously described [21]. Briefly, 2×10^13^ phage clones were first incubated with phosphate buffered saline (PBS) containing 2% nonfat dry milk (Carnation) for 30 min at room temperature (RT), to which 2×10^7^ yeast cells expressing unrelated protein (Zif268) was added for depletion of non-specific phage binders. The mixture of phage and yeast cells were then incubated for 1 h at RT with agitation. Non-binding phage clones in the supernatant were removed after centrifugation to pellet yeast cells, which were subsequently washed five times with PBS/0.05%Tween 20. Then phage particles were eluted from yeast cells with trypsin and used to infect *Escherichia coli* (TG1) to amplify enriched phage library for the next round of selection. After three rounds of selection, ∼200 TG1 clones were picked to produce individual phage clones. The binding of phage clones to ephrin-B2 expressing yeast cells was measured by immunofluorescence flow cytometry using anti-His tag antibody (Sigma-Aldrich) followed by goat phycoerythrin-conjugated anti-mouse antibody (Santa Cruz Biotechnology).

### Expression of scFv

Individual phage clones with high affinity binding to ephrin-B2 were sequenced, and selected scFv antibodies were solubly expressed from bacteria HB2151 (Clontech). Transformed HB2151 cells were grown at OD_600_ = 0.4, to which 1 mM isopropyl β-D-1-thiogalactopyranoside was added. Cells were induced to express protein at 25°C by culturing for 16 h with shaking at 250 rpm. After induction, cells were spun down, resuspended in binding buffer (50 mM sodium phosphate, pH 8.0, 300 mM sodium chloride, and 10 mM imidazole), sonicated to break the cell wall, and were spun at 10,000 g for 15 min to remove cell debris. Proteins in the supernatant were purified with Ni-NTA column followed by gel filtration chromatography using Superdex 75 column connected to AKTA Purifier (GE Healthcare). The purity and the size of proteins were confirmed by sodium dodecyl sulfate (SDS) polyacrylamide gel electrophoresis (PAGE).

### Cell culture

Chinese hamster ovary (CHO) cells [30] were maintained in Ham's F12 medium (Invitrogen) supplemented with 2 mM L-glutamine and 5% fetal bovine serum (FBS). Colorectal adenocarcinoma COLO205 [31] were cultured in RPMI 1640 medium supplemented with 2 mM L-glutamine, 10% FBS and 1% Penicillin-Streptomycin. HCT116 colon cancer cell line [32] was grown in McCoy's 5A Medium (Invitrogen) supplemented with 10% FBS. Human embryonic kidney (HEK) 293T cells [30] were maintained in advanced DMEM supplemented with 10% FBS, 2 mM L-glutamine, 20 µg/mL hygromycin and 1% Penicillin-Streptomycin. All cell cultures were maintained at 37°C in a humidified 5% CO_2_ atmosphere. 293T cells stably expressing ephrin-B2 were established by transfection of cells with pcDNA3.1 (Invitrogen) containing full-length ephrin-B2 with 200 µg/ml of hygromycin for selection.

### Immunofluorescence flow cytometry

Protein expression on yeast cell surface was analyzed by flow cytometry as described [33]. Briefly, yeast cells were incubated with primary antibodies (10 µg/ml) in 200 µl of labeling buffer (PBS/0.5% bovine serum albumin) for 1 h with shaking at 30°C. Cells were then washed and incubated with secondary antibodies in 200 µl of labeling buffer for 1 h at RT. After washing, cells were resuspended in 200 µl of labeling buffer and subjected to flow cytometry. The expression of ephrin-B2 in mammalian cells was detected by incubating cells with ephrin-B2 antibody for 1 h at 4°C, followed by Alexa Fluor 488 (AF488)-conjugated goat anti-human secondary antibody (Invitrogen). Flow cytometry was performed on a FACSCalibur (BD Biosciences) and analyzed with CellQuest software (BD Biosciences).

### Conversion of scFv into scFv-Fc

In order to fuse scFv into immunoglobulin G (IgG) constant region (Fc) including a hinge sequence, scFv sequence followed by IgG1 Fc was cloned into pcDNA3.1. scFv-Fc fusion protein was expressed in 293T cells and purified with Protein A beads (Thermo Scientific).

### Conformation specificity and selectivity of EC8 against ephrin-B2

Yeast cells expressing ephrin-B2 were incubated either in 50 mM Tris buffer (pH 8.0) containing 6 M guanidine hydrochloride for 20 min at room temperature, or in labeling buffer (PBS/0.5% bovine serum albumin) heated to 80°C for 10 min, to denature ephrin-B2. EC8 or EphB4-Fc binding to yeast cells after denaturation of ephrin-B2 was then measured by flow cytometry. To examine crossreactivity of EC8 to other ephrins, human ephrin-B1 (Leu-24 to Gly-165) and ephrin-A5 (Pro-22 to Gly-169) ectodomains were cloned and displayed on yeast surface. Immunofluorescence flow cytometry was conducted with mouse anti-Myc antibody (9E10, Santa Cruz Biotechnology), recombinant human EphB2/Fc chimera (R&D Systems), and EC8 followed by respective secondary antibodies as previously described.

### Competition assay

Yeast cells expressing ephrin-B2 were incubated with varying concentrations of mAb EC8 or affinity (protein A column, Pierce) purified human IgG for 20 min at 30°C, to which recombinant human EphB4 containing His-tag at C-terminal (R&D Systems) was added at 100 nM and incubated for 20 min at 30°C. The binding of EphB4 to yeast was detected by flow cytometry using anti-His tag antibody, followed by phycoerythrin-conjugated goat anti-mouse antibody.

### Immunoprecipitation of ephrin-B2 by mAb EC8

293T cells with stable expression of ephrin-B2 were incubated in lysis buffer (20 mM Tris-HCl, pH 8.0, 137 mM NaCl, 10% glycerol, 1% Triton X-100, 2 mM ethylenediaminetetraacetic acid (EDTA), protease inhibitor cocktail (Sigma)) for 30 min on ice. Cell debris were removed by centrifugation for 20 min at 14,000 g. Total protein lysate was precleared by Protein A beads for 1 h at 4°C. After centrifugation, supernatants were collected and incubated with 10 µg/ml EC8 for 2 h at 4°C, followed by incubation with Protein A beads overnight at 4°C. Immunoprecipitated proteins were run on SDS-polyacrylamide gel and transferred to polyvinyl difluoride membranes (Bio-Rad) for western blot analysis. The membrane was first blocked with 5% non-fat milk in TBS/0.05%Tween 20 for 1 h at RT, incubated with rabbit polyclonal anti-ephrin-B2 antibody (Genscript) overnight at 4°C, and detected by horseradish peroxidase (HRP)-conjugated anti-rabbit antibody (Santa Cruz). ECL substrate (Pierce) was added to the membrane for 1 min at RT, which was exposed to X-ray film.

### Immunofluorescence microscopy for ephrin-B2 labeling and internalization by EC8

Antibody labeling of ephrin-B2 was examined separately for cell-surface vs. internalized pool of ephrin-B2. 293T cells were grown on culture slides (BD Biosciences) to 50% confluence, to which 10 µg/ml of anti-ephrin-B2 antibody (EC8) was added. Cells were then incubated for 1 h at 37°C to allow antibody binding and endocytosis to occur. To detect EC8 bound to cell surface ephrin-B2, cells were fixed with 4% formaldehyde in PBS for 20 min at RT and incubated with blocking solution (PBS with 5% goat serum) for 1 h, followed by incubation with fluorescein-conjugated goat anti-human antibody (Invitrogen). Then to label internalized EC8, cells were permeabilized by 100% ice-cold methanol for 15 min on ice and incubated with blocking solution for 30 min at RT, followed by incubation with rhodamine-conjugated anti-human antibody (Invitrogen). Cell surface vs. internalized antibodies were imaged by confocal microscope (Zeiss LSM 710).

### RT-PCR

Total RNA was isolated from cultured cells with RNAeasy kit (Qiagen, Valencia, CA) according to the manufacturer's protocol. 1 µg of total RNA was used for reverse transcription. The expression of GAPDH and ephrin-B2 was analyzed with the following primers: GAPDH: 5′-TTGAGGTCAATGAAGGGGTC/5′-GAAGGTGAAGGTCGGAGTCA; Ephrin-B2: 5′-GTGTGGAAGTACTGCTGGGGTGTT/5′-GGCACAGTTGAGGAGAGGGGTATT.

### Immunohistology of tumor xenograft

10^6^ human colon cancer cells (COLO205 and HCT116; a kind gift from Dr. Michael King, Cornell University) were mixed in 0.1 ml 50% Matrigel (BD) and xenografted into nude mice (Jackson Laboratory). 14 days after injection when xenograft tumor size reached ∼80 mm^3^, mice were euthanized and tumors were harvested. All animal experiments were conducted in compliance with the regulations defined by the Institutional Laboratory Animal Use and Care Committee of Cornell University (Protocol number 2009-0071). Harvested tumors were fixed in 4% paraformaldehyde overnight at RT, rinsed 3 times for 10 min at RT each in 30% ethanol and then 50% ethanol, and then kept in 70% ethanol for several hours before embedding into a paraffin block for sectioning. In order to detect ephrin-B2 in the xenograft sections, tissues sections were deparaffinized in xylene 3 times for 4 min each at RT, rehydrated with graded ethanol, and then boiled in 10 mM citrate buffer (pH 6.0) for 10 min for antigen retrieval. The tissue sections were blocked with blocking solution (25 mM Tris-HCl, pH 7.4, 150 mM NaCl, 2 mM KCl, 0.3% Triton X-100, 1% goat serum) for 1 h at RT. Sections were incubated with the primary antibodies at 1 µg/ml overnight at 4°C, followed by incubation with secondary biotin-conjugated goat anti-human antibody (Invitrogen). Endogenous peroxidase activity was quenched with 0.3% hydrogen peroxide in methanol for 10 minutes at RT. Peroxidase-conjugated streptavidin (Vectastain Elite ABC stain, Vector Laboratories) was added to the sections. After each incubation, sections were washed 3 times with TBS buffer (25 mM Tris, pH 7.4, 150 mM NaCl, 2 mM KCl). Sections were then exposed to chromogen diaminobenzidine peroxidase substrate (Invitrogen) for 5 min, rinsed with water, and counterstained with Mayer's hematoxylin, dehydrated in graded ethanol, and cleared in xylene. Slides were sealed in mounting medium and a coverslip. Negative control slides were treated same way except for the use of primary antibody.

### Immunohistology of human tissue array

Human tumor tissue array (TP484, US Biomax) was deparaffinized and subjected to the same procedures as used for the immunostaining of tumor xenograft with EC8. Selected tumor tissue array slides were also used for immunofluorescence. After antigen retrieval and blocking, sections were incubated with primary antibodies (EC8, mouse anti-human CD31 antibody (Invitrogen)) at 1 µg/ml overnight at 4°C. After washing, tissue sections were incubated with AF488-conjugated goat anti-mouse secondary antibody, biotin-conjugated goat anti-human antibody, and were subsequently labeled with rhodamine-conjugated streptavidin. After each incubation, sections were washed 3 times with TBS buffer (25 mM Tris, pH 7.4, 150 mM NaCl, 2 mM KCl). Immunofluorescent images were acquired with a fluorescent microscope (MetaMorph, Molecular Devices).

### Statistical analysis

Data were expressed as mean ± standard deviation of no smaller than triplicates, and analyzed for statistical significance using GraphPad Prism 5 (Graphpad Software). Student's t-test was used to compare the binding levels and to determine statistical significance (Fig. 2D).
